# Prevalence, Knowledge, and Attitudes of Energy Drink Consumption Among University Students in the United Arab Emirates: A Cross-Sectional Study

**DOI:** 10.7759/cureus.83073

**Published:** 2025-04-27

**Authors:** Shooq Faqeeh, Saif Eddin Mansour, Raghd Darwish, Nafe Alhariri, Haya Alsebai, Khulood Alkalbani, Mohamed A Saleh, Amal Hussein

**Affiliations:** 1 Clinical Sciences Department, College of Medicine, University of Sharjah, Sharjah, ARE; 2 Family and Community Medicine Department, College of Medicine, University of Sharjah, Sharjah, ARE

**Keywords:** attitudes, consumption, energy drink, knowledge, prevalence, uae

## Abstract

Background

The consumption of energy drinks (EDs) among university students in the United Arab Emirates (UAE) has not been extensively studied. Therefore, this cross-sectional study aimed to investigate the prevalence, attitudes, and knowledge regarding EDs among university students in the UAE.

Objective

The primary objective of this study was to evaluate the prevalence, attitudes, motives, consumption patterns, and practices toward ED consumption among university students in the UAE. Furthermore, knowledge levels of ingredients and side effects were assessed.

Methods

This cross-sectional study was conducted via non-probability volunteer sampling to collect data from undergraduate university students in the UAE between February and April 2022. An online self-administered questionnaire was developed for data collection, and data were analyzed using IBM SPSS Statistics for Windows, version 28.0 (IBM Corp., Armonk, NY).

Results

A total of 471 participants completed the survey, of whom 355 (75.4%) were female. In all, 170 (36.1%) participants were current ED consumers, and taurine and caffeine-based EDs were the most commonly consumed type (n = 107, 62%). No participants displayed good knowledge of ingredients present in EDs; however, 234 (49.7%) demonstrated good knowledge of their potential side effects. Gender was significantly associated with the frequency of ED consumption (p = 0.027), and younger students (<18 years) were 2.36 times more likely to be consumers of EDs (confidence interval = 95%).

Conclusion

These results highlight insufficient knowledge and poor practices regarding proper ED consumption, despite a significant portion of university students being consumers. There is a need for interventions that increase awareness about the potential negative effects of ED consumption among university students in the UAE. Our findings also suggest that younger students may be more susceptible to consuming EDs, indicating a need for targeted interventions aimed at this population. Overall, this study provides valuable insight into the prevalence, attitudes, and knowledge of ED consumption among university students in the UAE.

## Introduction

EDs are over-the-counter, high-caffeine-containing beverages that are widely known to boost physical performance, alertness, reduce fatigue, and enhance energy levels [1]. In recent years, there has been a marked increase in the prevalence of consumption of EDs among young adults [2-4], contributing to more than half of the consumer market worldwide [5,6]. More than half of college students in a research article agreed that EDs are a good source of energy [7]. One of the main reasons to consume EDs was to stay awake, for study purposes, and [5] to enhance academic performance [7].

Common ingredients of EDs include caffeine, taurine, ginseng, guarana, B vitamins, etc. [1,3,4]. EDs can have an energizing outcome that reaches a climax after 30-60 minutes of consumption, aligning with the plasma peak of glucose and caffeine, and the effect wears off after 90 minutes upon utilization by 18-55 age groups [5,8].

Adverse effects experienced by consumers include insomnia, anxiety, agitation, chest pain, and palpitations [9-11]. As caffeine is one of the predominant stimulatory components in EDs, consuming it in a moderate dose level of 400 mg of caffeine per day is not associated with its adverse effects and can prevent several diseases [12-14]. However, consuming EDs in excess can lead to addiction and, in some cases, can potentially precipitate intoxication [15,16]. In all, 100 mL of ED contains 80-242 mg of caffeine, which is proportionate to eight strong cups of coffee [4,17]. The acute heavy consumption of EDs has led to arrhythmias, ST-segment elevation, and cardiac arrest [18], also associated with a lethal dose of 5 g of caffeine [12]. Almost half of the patients had cardiac outcomes, mainly arrhythmias, and others had neurological, gastrointestinal, renal, dermatological, and gynecological outcomes [19]. Sudden death has been reported in healthy individuals who are known to consume caffeinated drinks [20].

A review of the literature finds limited studies on energy drink (ED) consumption in the United Arab Emirates (UAE). The study aims to assess the prevalence, attitudes, motives, practices toward ED consumption, and knowledge levels of ingredients and side effects among university students in the UAE in a broader scope. Secondarily, the study aims to evaluate the effect of various demographic factors on consumption patterns and knowledge levels.

## Materials and methods

Study design and setting

A cross-sectional study was conducted from February to April 2022. We used a cross-sectional study design as we did not aim to collect serial measurements, and the study design was most suitable to measure the prevalence, knowledge, and attitudes of ED consumption among university students. An online questionnaire was administered and shared with university students throughout the UAE via direct messages and social media platforms, including WhatsApp, Instagram, and Telegram. The Strengthening of the Reporting of Observational Studies in Epidemiology (STROBE) checklist was used in reporting this study [21].

Ethical considerations

This study was approved by the Research & Ethics Committee (REC) at the University of Sharjah, Sharjah, UAE (REC-22-02-14-04-S), and a Participant Information Sheet (PIS) was displayed prior to participation to grant consent.

Study population and sampling method

This study targeted undergraduate university and college students in the UAE. A non-probability volunteer sampling method was used to recruit participants, which may have led to selection bias. To counteract this, recruitment was conducted across various diverse social media platforms in order to capture a more representative sample.

Participants were required to meet specific inclusion and exclusion criteria to ensure the relevance of the study findings. Undergraduate university students who were English or Arabic speakers were incorporated into the study. In contrast, students who were not willing to participate in the questionnaire, students who were not present in the UAE, and students who did not complete the questionnaire were excluded.

The needed sample size was calculated using Cochran’s formula: n = 1540(P)(1 − P), which is determined by the expected prevalence of the condition being studied [22,23]. Where “n” is the minimum sample size, and “P” is the expected prevalence. The “P” value was obtained from two similar articles in the Middle East and North Africa (MENA) region that found a prevalence of approximately 30% [24,25]. A level of precision set at 5% and a level of confidence interval (CI) of 95% were used. Therefore, to achieve a precision of ±5% with a 95% CI and P = 0.30, the sample size was 329, which was exceeded by 10%, reaching 362 to account for missing data.

Questionnaire development and data collection

Due to the lack of existing tools to assess attitudes toward ED consumption in the UAE, an anonymous and confidential online self-administered questionnaire was developed (see Table 3 in Appendices). The questionnaire consisted of four sections and 25 items in both Arabic and English versions. The four sections inquired about sociodemographic characteristics, history of ED use, justification of consumption, awareness of ingredients, and health risks associated with consumption. The participants were provided with electronic informed consent before proceeding. 

Some of the questionnaire items were adopted from other articles with previously validated questionnaires that were found to be relevant to the study [3,26], and to fulfill our research objectives, a few items of our own were developed. Most questions were closed-ended, and others were multiple-choice questions with some of them being multiple-select answers, in addition to one rating scale question.

Variables and measurements

The study examined various variables that were divided into dependent and independent categories to assess factors influencing ED consumption among university students in the UAE. The independent variables included demographic characteristics (age, gender, emirate of residency, and living status) and academic factors, including year of study and major. Dependent variables included knowledge-related factors regarding ED ingredients and potential side effects, satisfaction after consumption, beliefs of substitute drinks, consumption, frequency, and type of ED consumed.

The knowledge scores were developed based on how many ingredients or side effects the participants were able to identify from the options listed in the question. Each correct response contributed to their total knowledge score, which was analyzed as a continuous variable. The options were adapted from the certified ingredients written on the label of most EDs and the scientifically proven side effects [1,19,27]. The knowledge scores were categorized as poor (score of less than 3), average (score of 3), and good (score of more than 3). 

Validity

To ensure content validity of the questionnaire, the questions were designed to adequately cover all key aspects of ED knowledge, consumption, and attitudes. They were adapted from existing literature and designed to meet our study objectives. Furthermore, experts and researchers in the field of pharmacology were consulted to verify the questionnaire items and their relevance, alongside researchers in the field of family and community medicine, guaranteeing face validity through active and continuous feedback and supervision throughout the development of the questionnaire. 

A pilot study was conducted to test the clarity, reliability, and relevance of the questionnaire before distributing it to the full sample population. The aim was to identify and correct any vague or confusing questions or options in the questionnaire and ensure the overall quality of the target population. The pilot study was carried out among 32 university students who fell under the inclusion criteria. These participants were not included in the final analysis. After completing the questionnaire, participants were asked to provide feedback on the wording, length, and clarity of the questions. After reviewing the feedback, minor changes were made to the wording and question structure to improve comprehension and relevance. This process helped enhance the face validity of the instrument and ensured that it was suitable for the larger sample.

Data analysis

The data were cleaned and analyzed using IBM SPSS Statistics for Windows, version 28.0 (IBM Corp., Armonk, NY). Descriptive statistics were used to summarize the demographics and knowledge score. Frequencies and percentages were reported for categorical variables. Fisher’s exact test was used for bivariate correlation when the expected values in the analysis were less than 5; otherwise, chi-square (Χ²) was used for bivariate analysis. The odds ratio (OR) and 95% CI were calculated. The level of significance for the p-value was set at 5%.

## Results

Study demographics

This study included a total of 471 university students across 29 different academic majors. The sample consisted of 116 males (24.6%) and 355 females (75.4%), resulting in a male-to-female ratio of 1:3.06. Among the participants, 247 students (53.7%) were enrolled in medical colleges, while 213 students (46.3%) were from non-medical majors (Table 1).

**Table 1:** Demographics of study participants

Demographics	n (%)
Age	
<18 years	43 (9.1%)
18-21 years	375 (79.6%)
≥22 years	53 (11.3%)
Gender	
Male	116 (24.6%)
Female	355 (74.4%)
Major	
Medical colleges	247 (52.4%)
Non-medical colleges	213 (45.2%)
Year of study	
Foundation year	40 (8.5%)
Year 1	97 (20.6%)
Year 2	156 (33.1%)
Year 3	104 (22.1%)
Year 4/5	74 (15.7%)
Living status	
Dormitory living	75 (15.9%)
Living with family	381 (80.9%)
Living alone	15 (3.2%)
Emirate of residency	
Abu Dhabi	119 (25.3%)
Dubai	77 (16.3%)
Sharjah	210 (44.6%)
Ajman	39 (8.3%)
Ras Al Khaimah	12 (2.5%)
Fujairah	6 (1.3%)
Umm Al Quwain	8 (1.7%)

Prevalence of ED consumption

**Figure 1:**
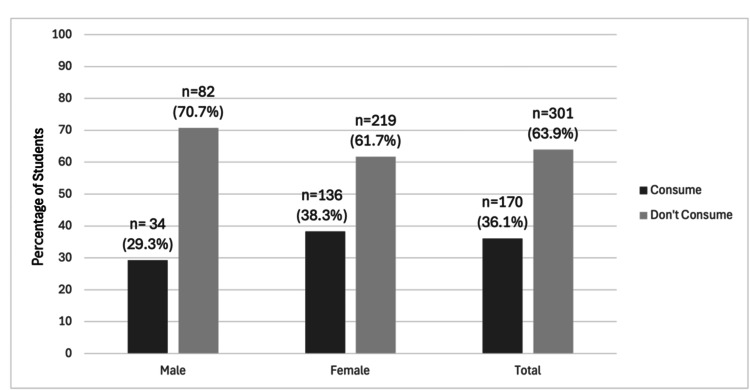
Prevalence of energy drink consumption among university students

The total number of university students who consumed EDs was 170 (36.1%). The prevalence was slightly higher among females (136 students, 38.3%) compared to males (34 students, 21.3%), though this difference was not statistically significant. A statistically significant number of males were found to consume EDs more frequently when compared to females (29.4% of males & 17.6% of females consume four or more cans weekly, p = 0.027). Regarding age groups of consumers, a significantly higher proportion of students <18 years (n = 23, 53.5% of all students aged <18 years) consumed EDs when compared to other age groups (n = 123, 32.8% of those aged 18-21, and n =24, 45.3% of those aged ≥22 years, p = 0.009), and it was found that students younger than <18 years of age were 2.36 (CI = 95%) times more likely to be current consumers of EDs (Figure 1).

**Figure 2:**
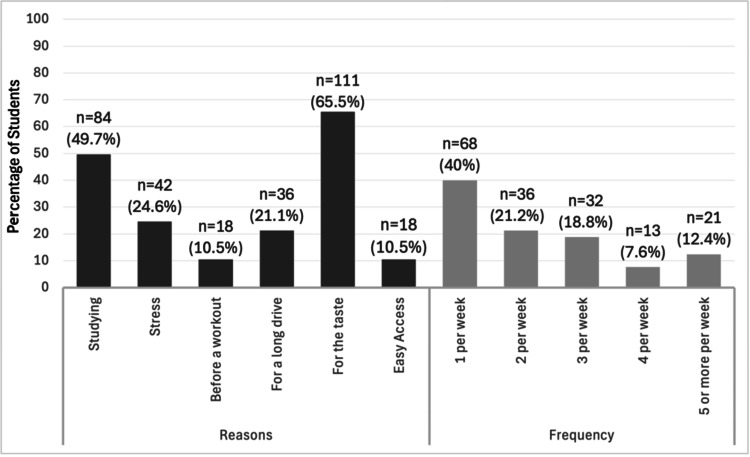
Reasons for and frequency of energy drink consumption

The most consumed type of ED was taurine and caffeine-based ED (n = 107, 62.4%), followed by vitamin-enriched ED (n = 31, 18.2%), and then caffeine-enriched sports ED (n = 11, 6.5%). The most common motivations for consuming EDs were enjoying the taste (n = 111, 65.5%), studying (n = 84, 49.7%), stress (n = 42, 24.6%), and long drives (n = 36, 21.1%). Regarding frequency of consumption, 68 (40%) of the current users consumed one can per week, 36 (21.2%) consumed two cans per week, 32 (18.8%) consumed three cans per week, and 34 (20%) drank four or more cans per week (Figure 2).

The relationship between ED consumption and the different studied variables among university students was analyzed. The prevalence of ED consumption was comparable between medical and non-medical students, where 87 (35.2%) medical and 77 (36.2%) non-medical students were current consumers; however, no statistical significance was found (p > 0.05). Additionally, ED consumption was most prevalent among foundation-year students (n = 20, 50%) and second-year students (n = 62, 39.7%), though the differences were not statistically significant (p > 0.05) (Table 2).

**Table 2:** Relationship between energy drink consumption and different studied variables among university students *p < 0.05

Variables	Energy drink consumption		Test statistic	p-value
	Yes (%)	No (%)		
Gender				
Male	34 (29.3%)	136 (38.3)	χ2 = 3.070	p = 0.08
Female	82 (70.7%)	219 (61.7)		
Age group				
<18 years	23 (53.5)	20 (46.5)	χ2 = 9.345	p = 0.009*
18-21 years	123 (32.8)	252 (67.2%)		
≥22 years	24 (45.3)	29 (54.7)		
Year of study				
Foundation year	20 (50)	20 (50)	χ2 = 7.347	p = 0.119
Year 1	31 (32)	66 (68)		
Year 2	62 (39.7)	94 (60.3)		
Year 3	30 (28.8)	74 (71.2)		
Year 4/5	27 (36.5)	47 (63.5)		
Major				
Medical colleges	87 (35.2)	160 (64.8)	χ2 = 0.043	p = 0.863
Non-medical colleges	77 (36.2)	136 (63.8)		
Emirate of residency				
Abu Dhabi	45 (37.8)	74 (62.2)	χ2 = 3.736	p = 0.291
Dubai	33 (42.9)	44 (57.1)		
Sharjah	74 (35.2)	136 (64.8)		
Other Emirates	18 (27.7)	47 (72.3)		
Living status				
Dormitory living	26 (34.7)	49 (65.3)	χ2 = 6.285	p = 0.043*
Living with family	134 (35.2)	247 (64.8)		
Living alone	10 (66.7)	5 (33.3)		

Knowledge of EDs’ ingredients and side effects

**Figure 3:**
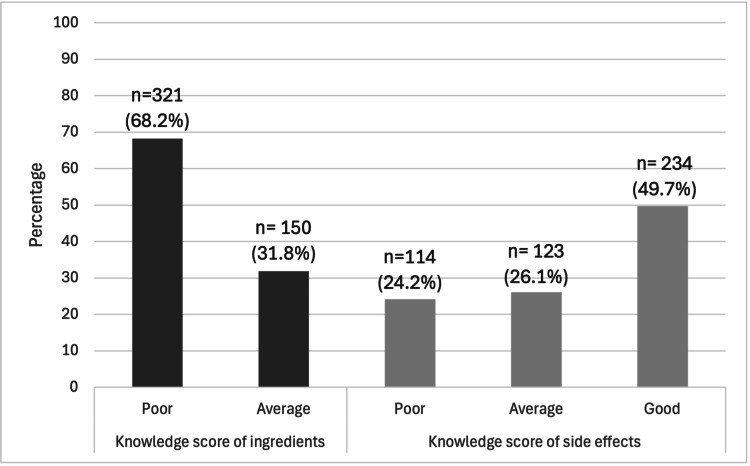
Knowledge scores of ingredients and side effects

Participants’ knowledge of ED ingredients and their associated side effects was assessed. None of the students achieved a good knowledge score regarding the ingredients present in EDs; however, 234 (49.7%) participants displayed a good knowledge score of their potential side effects. Females were significantly more likely to achieve a good knowledge score of side effects when compared to males, as 187 (52.7%) of females demonstrated good knowledge of side effects, whereas 47 (40.5%) of males displayed the same knowledge level (p = 0.026) (Figure 3).

A notable difference was observed among students’ knowledge from different emirates, where residents of Abu Dhabi were significantly more likely to have a poor knowledge score of ED ingredients compared to students from other emirates, as 91 (76.5%) of Abu Dhabi residents demonstrated poor knowledge of ingredients (p = 0.017).

## Discussion

EDs are emerging as a public health threat, as their marketing has been exponentially growing over the past decade and a half, leading to an increase in consumption among adolescents internationally [28,29]. Our study, which employed an online questionnaire distributed among university students through social media, provides valuable insight into the knowledge, attitudes, and practices toward EDs among university students in the UAE. More than one-third of our participants were current consumers of EDs. Despite this, the findings indicate a significant lack of knowledge concerning the side effects and the content of EDs. Attitudes toward ED consumption varied, and practices of current consumers were found to be lacking.

Other studies done in the region show similar prevalence and patterns of ED consumption among university students. A study done in Saudi Arabia by Bawazeer and AlSobahi and another by Katib et al. showed a similar prevalence of ED consumption among university students of around one-third [24,30]. On the other hand, similar studies done in the UAE, utilizing a similar methodology of self-administered questionnaires, previously showed that 85.1% and 92% of university students consume EDs [31,32]. Thus, it is evident that the use of EDs in our study is lower compared to previous ones done in the UAE, possibly due to the recent excise tax of 100% that was implemented on EDs in 2017 [33].

Our findings demonstrated no significant discrepancy in the prevalence of ED consumption among females and males, unlike other studies that showed that males were significantly more likely to be consumers of EDs [30]. Despite this, higher rates of consumption were observed among males included in the study, which is possibly attributable to various factors involving the fact that these drinks are advertised to provide energy to accomplish physically and mentally demanding activities, which may be attractive for young male adults, as suggested by Chiou et al. [34]. However, differences in the total sample numbers of males and females (355 females vs. 116 males) may have introduced some bias.

In our study, younger university students were found to be more prevalent consumers of EDs, while other studies showed a more even distribution of consumers based on age [3,24]. This variation in results could be attributed to the stress of the transitional period between school and university. It was initially hypothesized that medical students would use EDs more frequently than students from other majors. However, we did not find a significant difference between the different majors, in contrast with other studies, which found that students in science colleges were more likely to be consumers of EDs than those in health-related colleges (89.4% versus 74.4%, respectively) [35].

The motivation to consume EDs for energy or while studying may be attributable to the effects of their caffeine content, as caffeine blocks adenosine receptors in the nervous system. This action inhibits the activity of neurotransmitters that promote relaxation and sleepiness and increases the release of other neurotransmitters, including norepinephrine and dopamine, which help enhance energy levels, focus, and mood [36]. This aligns with our findings, where a substantial proportion of participants in our study consumed EDs for the taste and while studying. These results are consistent with previous studies [31], with the main reasons for consumption being taste (39.9%), energy needs (27.7%), and while studying (13.1%). Other motivations for consuming EDs in other regions of the world are consuming these beverages mixed with alcohol to be inebriated, reducing the sedating effects of alcohol when drunk alone, and adding a new flavor to an alcoholic mixture [37]. In the UAE, alcohol consumption is allowed in specific contexts and venues. Given the cultural sensitivity aspect, our study did not investigate the mixing of alcohol with EDs.

EDs are widely consumed, particularly among young adults, and typically contain ingredients such as caffeine, taurine, guarana, and sugar, all of which have been linked to various adverse health effects [1]. Despite these risks, our study revealed a significant gap in awareness: 0% of participants demonstrated good knowledge of ED ingredients. Furthermore, over half of the students had only average or poor knowledge regarding their side effects. Similar findings have been reported in a study from Saudi Arabia, where only a few participants were aware of the composition of EDs [38]. A study done in Turkey by Attila and Çakir showed similar results, where most students could not correctly identify the ingredients of EDs and their potential hazardous side effects [39]. This widespread lack of knowledge is concerning, particularly given the easy accessibility and aggressive marketing of EDs to youth. Addressing this gap through targeted education is essential to mitigate the health risks associated with their frequent and uninformed consumption. 

The results of our study have crucial regulatory and clinical consequences, acknowledging the high caffeine content of EDs along with the marketing strategies targeting adults and youths with limited educational backgrounds. This study provides a clear picture of the usage, practices, and education levels among university students in the UAE. There is an ever-growing theoretical and evidence-based support for a link between ED consumption and serious health-related issues, including cardiovascular and neurological adverse effects [9,40].

It is important to note that the cross-sectional design of our study and the use of non-random sampling may have introduced selection bias. Furthermore, our study did not assess the side effects that were experienced by consumers and was directed toward university students rather than the general population in the UAE. Confounding factors such as demographics were collected through the questionnaire; however, these were not specifically addressed in the analysis. Despite these limitations, our questionnaire has been culturally adapted and piloted for clarity, along with adherence to ethical and STROBE guidelines, which ensures the reliability of collected data and reproducibility of results based on the used methods. Future studies could focus on exploring the cause-and-effect relationship by using randomized sampling methods in order to reduce selection bias or clinical trials that aim to study causation between variables. 

This study highlights the importance of implementing awareness campaigns and educational programs targeting adolescents and young adults. Such actions aim to correct common misconceptions and educate individuals about the ingredients, potential side effects, and safe consumption of EDs. Given that all participants in this study reported using social media, incorporating such educational programs into mobile applications could significantly enhance the engagement of the target population. Research has found that when EDs are promoted online, young adults are more likely to consume them, highlighting the importance of using the same platforms to promote awareness against excessive consumption of these drinks [41].

In addition to awareness, interventions should be implemented as policies that require ED manufacturers to provide clear labeling of active ingredients and their associated health risks, similar to how tobacco products are labeled. Such policies have been shown to increase consumer awareness, reduce usage, and control excessive consumption, especially in younger individuals [42]. Interestingly, young people seem to understand the importance of spreading awareness about this topic. In one study, many participants supported interventions such as school programs, better health messages, and clear public information to help people understand the risks and oppose the marketing approach [43]. This shows that while education is essential, it may not suffice alone. If we want to make a difference, we need to combine awareness with real policies that are strictly implemented. Implementing education and regulation could be the most effective and long-term way to reduce the harm caused by EDs.

## Conclusions

Our study reveals that more than one-third of university students in the UAE are current consumers of EDs. Yet, none of the participants were aware of the side effects, and the majority displayed poor knowledge of ingredients. Along with the continuous growth of marketing campaigns and consumption of EDs comes a need to improve education and outreach regarding this topic. To address the subject, awareness campaigns and educational programs should focus on improving knowledge, attitudes, and safe ED consumption practices. Given the high level of outreach that can be accessed through social media, educational videos or interactive posts can be used to enhance accessibility and engagement. Future research should focus on measuring the side effects on consumers and the effect of awareness campaigns on university students and the general population in the UAE.

## Appendices

**Table 3:** English version of the questionnaire

Category	Question	Answer options
Willingness to participate	W. Are you willing to participate in this survey?	Yes
		No
Demographics	D1. What is your age in years?	<18 years
		18-21 years
		22-25 years
		>25 years
	D2. What is your gender?	Male
		Female
	D3. What is your college major?	Medicine
		Pharmacy
		Dentistry
		Health Sciences
		Engineering
		Fine Arts
		Arabic Literature
		Other: specify
	D4: Which year of study are you in?	Foundation year
		Year 1 (freshman)
		Year 2 (sophomore)
		Year 3 (junior)
		Year 4/5 (senior)
	D5: What is your living status?	Dormitory living
		Living with family
		Living alone
		Other: specify
	D6: In which Emirate do you live?	Abu Dhabi
		Dubai
		Sharjah
		Ajman
		Ras Al Khaimah
		Fujairah
		Umm Al Quwain
Attitudes	A1: Do you currently consume any energy drinks?	Yes
		No (skip to A8)
	A2: Which energy drink have you consumed most frequently?	Taurine and caffeine-enriched energy drinks
		High caffeine performance energy drink
		Vitamin-enriched energy drink
		Ginseng-infused energy drink
		Caffeine-enriched sports energy drink
		Herb-containing energy drink
		Other: specify
	A3: Have you ever combined more than one energy drink?	Yes
		No
	A4: How many cans of energy drinks do you drink per week?	1
		2
		3
		4
		5 or more
	A5: What is your main reason for consumption of energy drinks? You can choose more than one.	Studying
		Stress
		Before a workout/sport
		For a long drive
		You like the taste
		Easy access
		Other: specify
	A6: When do you mostly consume energy drinks?	In the morning
		During the day
		At night
	A7: How much would you say your energy levels increased after consuming energy drinks?	Likert Scale 1 = not, 2, 3, 4, 5 = greatly increased
	A8: Which of the following is the reason you do not consume energy drinks? You can choose more than one.	I don’t like the taste
		It causes weight gain
		They cause health problems
		They are expensive
		I can’t access them
		Other: specify
Knowledge	K1: From where do you get information about energy drinks? You can choose more than one.	Family or friends
		Nutritionist or dietitian
		The Internet
		Advertisements
		Nutritional facts on the can
		Family or friends
		Other: specify
	K2: Which of these would you think is an ingredient of a typical energy drink? You can choose more than one.	Caffeine (a crystalline compound that is found especially in tea and coffee plants and stimulates the body)
		Sugar
		Vitamins
		Ginseng (a plant commonly known for its antioxidant and anti-inflammatory effects)
		Taurine (an artificial chemical substance utilized by the body during exercise and in times of stress)
		I don’t know
	K3: Which of these do you think can be the benefits of energy drink consumption? You can choose more than one.	Ability to stay awake longer
		Increased alertness
		Improvements in mental performance
		Increased power in exercise/sport
		Improves mood
	K4: Do you believe that consuming energy drinks causes the following health problems?	Select: Yes, no, I don’t know
	Heart problems	
	High blood pressure	
	Mental problems	
	Weight gain	
	Tooth cavities	
	K5: Do you think the benefits of energy drink consumption are greater than the risks?	Yes
		No
		Maybe
Beliefs	B1: Why do you think people use energy drinks? You can choose more than one.	Need more energy
		Studying/completing a major project
		Driving for a long time
		While partying
		Weight loss
		Athletic performance
		Refreshment/taste
		Relieve stress
		Easy access
	B2: Do you believe there are any substitutes for energy drinks? If yes, please specify using the "Other" section.	Yes
		No
		Other: specify
	B3: Do you think consuming energy drinks affects the person financially?	Yes
		No
	B4: If you are an energy drink consumer, do you think the recent increase in prices of energy drinks affected your consumption habits?	Yes
		No
	B5: If you are an energy drink consumer, would you recommend your friends/family members to try energy drinks?	Yes
		No
	B6: Are you interested in knowing about the dangers of energy drinks?	Yes
		No
